# HLA Genes, Islet Autoantibodies and Residual C-Peptide at the Clinical Onset of Type 1 Diabetes Mellitus and the Risk of Retinopathy 15 Years Later

**DOI:** 10.1371/journal.pone.0017569

**Published:** 2011-03-11

**Authors:** Richard A. Jensen, Elisabet Agardh, Åke Lernmark, Soffia Gudbjörnsdottir, Nicholas L. Smith, David S. Siscovick, Carina Törn, on behalf of the DISS Group

**Affiliations:** 1 Cardiovascular Health Research Unit, School of Medicine, and the Department of Epidemiology, School of Public Health, University of Washington, Seattle, Washington, United States of America; 2 Department of Clinical Sciences, Ophthalmology, University Hospital MAS, Malmö, Sweden; 3 Department of Medicine, University of Washington, Seattle, Washington, United States of America; 4 Department of Clinical Sciences, Clinical Research Center, Lund University, Malmö, Sweden; 5 Department of Medicine, Sahlgrenska University Hospital, Göteborg University, Göteborg, Sweden; 6 Seattle Epidemiologic Research and Information Center of the Department of Veterans Affairs, Office of Research and Development, Seattle, Washington, United States of America; 7 Group Health Research Institute, Group Health Cooperative, Seattle, Washington, United States of America; La Jolla Institute of Allergy and Immunology, United States of America

## Abstract

**Aims/Hypothesis:**

HLA genes, islet autoantibodies and residual C-peptide were studied to determine the independent association of each exposure with diabetic retinopathy (DR), 15 years after the clinical onset of type 1 diabetes in 15–34 year old individuals.

**Methods:**

The cohort was identified in 1992 and 1993 by the Diabetes Incidence Study in Sweden (DISS), which investigates incident cases of diabetes for patients between 15 and 34 years of age. Blood samples at diagnosis were analyzed to determine HLA genotype, islet autoantibodies and serum C-peptide. In 2009, fundus photographs were obtained from patient records. Study measures were supplemented with data from the Swedish National Diabetes Registry.

**Results:**

The prevalence of DR was 60.2% (148/246). Autoantibodies against the 65 kD isoform of glutamate decarboxylase (GADA) at the onset of clinical diabetes increased the risk of DR 15 years later, relative risk 1.12 for each 100 WHO units/ml, [95% CI 1.02 to 1.23]. This equates to risk estimates of 1.27, [95% CI 1.04 to 1.62] and 1.43, [95% CI 1.06 to 1.94] for participants in the highest 25^th^ (GADA>233 WHO units/ml) and 5^th^ percentile (GADA>319 WHO units/ml) of GADA, respectively. These were adjusted for duration of diabetes, HbA_1c_, treated hypertension, sex, age at diagnosis, HLA and C-peptide. Islet cell autoantibodies, insulinoma-antigen 2 autoantibodies, residual C-peptide and the type 1 diabetes associated haplotypes DQ2, DQ8 and DQ6 were not associated with DR.

**Conclusions:**

Increased levels of GADA at the onset of type 1 diabetes were associated with DR 15 years later. These results, if confirmed, could provide additional insights into the pathogenesis of the most common microvascular complication of diabetes and lead to better risk stratification for both patient screenings and DR treatment trials.

## Introduction

The World Health Organization estimates that more than 180 million people worldwide have diabetes mellitus and this number is likely to more than double by 2030; about 10% have type 1 diabetes mellitus [1]. Severe visual impairment develops in 10% of patients and 2% will be blind within 15 years of diagnosis [1]. Blood glucose control has been identified as a critical risk factor in the development and progression of diabetic retinopathy (DR) [2], [3] but does not completely explain the pathogenesis [4], [5]. In this study we hypothesize that autoimmune processes resulting from HLA genotype and the relationship of these genes with islet autoantibody status and residual C-peptide production at the clinical onset of diabetes are associated with the risk of DR 15 years later.

Type 1 diabetes begins as an autoimmune process that can be differentiated from type 2 diabetes by the presence of islet autoantibodies before [6], [7], [8] and at the time of clinical onset [9], [10]. These include islet cell autoantibodies (ICA) [11], [12], [13] and autoantibodies against specific autoantigens including the 65 kD isoform of glutamic acid decarboxylase (GADA) [14], [15], [16], insulinoma-antigen 2 (IA-2A) [17], [18], [19], insulin (IAA) [20], and the cation efflux transporter ZnT8 (ZnT8A) [21]. The presence of these islet autoantibodies is associated with genes in the HLA complex on chromosome 6, whether they occur alone [22] or with type 1 diabetes [23], [24], [25]. The two major risk haplotypes include DQ2 (DRB1*0301-DQA1*0501-B1*0201) and DQ8 (DRB1*04-DQA1*0301-B1*0302) and before the age of 15 years, DQ6 (DRB1*1501-DQA1*0102-B1*0602) is a protective haplotype [26]. Insulin secretion, measured by serum C-peptide, is severely impaired at the time of diagnosis of type 1 diabetes. There is typically a continuous decline as the disease progress [27] which is associated with the number and types of islet autoantibodies present [28].

The HLA gene complex has been repeatedly studied for its association with DR for the past 30 years with both negative [29], [30], [31], [32], [33], [34], [35], [36] and positive findings [37], [38], [39], [40], [41], [42], [43], [44], [45], [46], [47], [48], [49]. Two separate properties of the HLA complex make it difficult to study. It is polygenic as it contains several different MHC class I and MHC class II genes and it is the most polymorphic human gene known with hundreds of variants for some of these genes [50]. These properties make it difficult to interpret the results of these studies as they are hindered by small samples sizes in numerous comparison groups or have little information about other known risk factors for DR such as blood glucose control and hypertension. Unlike HLA, there have been few studies of islet autoantibodies or C-peptide and DR. Two small cross-sectional studies have reported an inverse relationship between levels of GADA and the severity of DR suggesting that GADA may inhibit one or more mediators of DR [51], [52]. In the Diabetes Control and Complications Trial, any C-peptide secretion, but especially higher and sustained levels of stimulated C-peptide, was associated with reduced incidences of DR [53].

**Figure 1:**
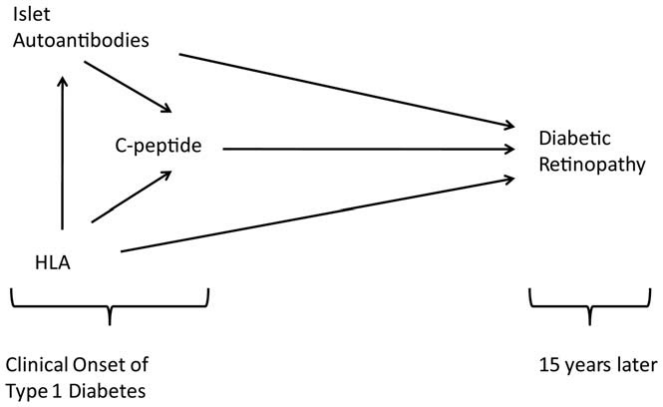
Study design showing the relationship between human leukocyte antigen genes, islet autoantibodies, C-peptide and diabetic retinopathy.

Previous studies have examined the cross-sectional associations of HLA, islet autoantibodies and residual C-peptide with DR; however, none of these studies has accounted for the relationships between these immunologic markers (Figure 1) to determine the independent association of each exposure with DR. This incident inception population-based cohort study uniquely uses measures of islet autoantibodies and C-peptide determined at the clinical onset of diabetes while limiting the testing of associations between HLA and DR to the three haplotypes (DQ2, DQ8 and DQ6) most closely related to type 1 diabetes.

## Methods

### Study Population

Written consent was obtained from all participants. The regional Ethics Board of Lund University, Lund, Sweden, approved the study.

The cohort for the present study was identified by the Diabetes Incidence Study in Sweden (DISS) during 1992 and 1993 [54]. DISS is an on-going prospective study that attempts to enroll all incident cases of diabetes for patients between the ages of 15 and 34 years. Ascertainment in the DISS study has been previously estimated at 86% for type 1 diabetes and 53% for type 2 diabetes [55]. Starting in 1992, participants provided blood samples at diagnosis and each year thereafter for 6 years to determine their levels of serum C-peptide and islet autoantibodies including ICA, GADA, IA-2A and IAA. ZnT8A were not described until 2007 [21] and were not analyzed.

A control group [56] of subjects without diabetes were matched by age and sex as cases were identified by DISS. There were slightly more controls than cases as some cases were later excluded when it was determined they did not have diabetes or had gestational diabetes. The control group provided a reference to determine the distribution of autoantibody and C-peptide levels in healthy non-diabetic subjects. This study preceded the Diabetes Autoantibody Standardization Program and World Health Organization's (WHO) standardization of diabetes autoantibodies so these controls provided the means to determine cut-offs for autoantibody positive status. Levels of autoantibodies in the present study have been converted to WHO International units (GADA, IA-2A) or Juvenile Diabetes Foundation Units (ICA). The control group was not used in analyses for the present study.

**Figure 2:**
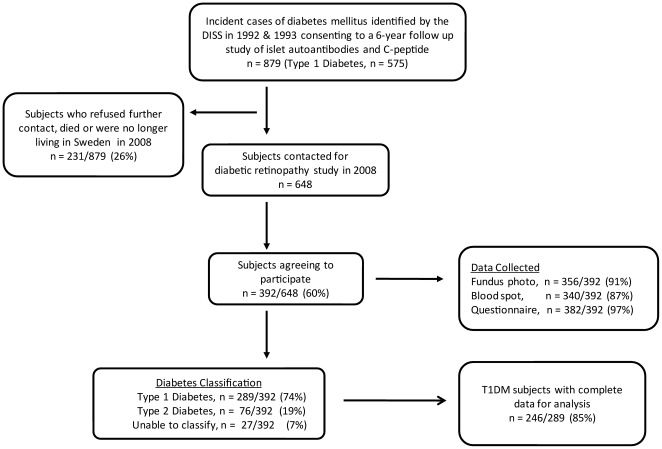
Flow diagram of study participants.

In 2008, we contacted 648 individuals to ask them to participate in this study (Figure 2). Current addresses were obtained from the Swedish Population and Address Register. The initial mailing included a questionnaire and a kit to collect a dried capillary blood spot. Individuals were contacted twice by mail and those who did not respond to either mailing received a phone call inviting them to participate in the study. The participation rate was 60% (392/648), of these, 74% (289/392) were classified with type 1 diabetes.

### HLA Genotyping

HLA genotyping for DRB1, DQA1 and DQB1 was carried out by PCR amplification of the second exon of the IDDM1 genes followed by dot blot hybridizations of sequence specific oligo probes and by restriction fragment length polymorphism using DR- and DQ-based probes to establish haplotypes [24]. In addition, allele specific PCR amplification of DRB1 alleles was also used [57], [58]. The haplotypes were classified as DQ2 (DRB1*0301-DQA1*0501-B1*0201), DQ8 (DRB1*04-DQA1*0301-B1*0302), DQ6 (DRB1*1501-DQA1*0102-B1*0602), or ‘other’, where other is not DQ2, DQ8, or DQ6.

### Islet Autoantibodies

The determination of autoantibody levels along with the sensitivity and specificity of our assays have been previously described [28]. Briefly, positive values for GADA and IA-2A, were determined using a cutoff of >97½ percentile of the values defined by a matched control group of 829 individuals [56]. GADA and IA-2A levels were measured by radioimmunoassay [59], [60] and expressed as an index [cpm of tested sample - average cpm of two negative standards] divided by [cpm of positive standard - average cpm of two negative standards]. IAA levels were also measured by radiobinding assay [20]. The IAA assay measures the percentage of displacement of the binding of radioactive insulin. A participant was considered to have type 1 diabetes if the displacement was >0.7% based on previous results from healthy individuals [61]. ICA levels were determined by standard immunofluorescence methods as previously described [62], [63]. Participants were considered to have type 1 diabetes if they tested positive for any of the four autoantibodies at the baseline exam; GADA>21.2 WHO units/ml, IA-2A>5.88 WHO units/ml, IAA>0.7% and ICA>6 Juvenile Diabetes Foundation Units. In the first Diabetes Autoantibody Standardization Program, sera from selected individuals were used to compare assay results from participating labs. This GADA assay had 80% sensitivity and 96% specificity, and the IA-2A assay had 58% sensitivity and 100% specificity [64]. The sensitivity of the ICA assay used in this study was 100% and specificity 88% for the pancreas when tested in the International Diabetes Workshop for standardization [65]. For this study, we considered the first blood draw after diagnosis to represent a baseline measure with the exception of IAA which needed to be completed within 1 month.

### C-peptide

C-peptide levels were determined at the Department of Clinical Chemistry, Skåne University Hospital SUS, Lund, Sweden using the EURIA-C-PEPTIDE kit MD315 (EuroDiagnostica, Medeon, Malmö, Sweden). By this method, the lower detection limit for C-peptide is 0.13 nmol/l. The 2.5^th^ percentile of C-peptide for the matched control group was 0.24 nmol/l [56].

### Questionnaire

The study questionnaire asked participants for the name of the clinic they visited during their most recent eye exam. The health history portion included the following questions: 1) Have you ever been told you have hypertension (HTN), impaired kidney function or increased cholesterol or triacylglycerol by a doctor or nurse? 2) Have you ever been prescribed medication to control HTN or cholesterol? 3) Are you currently using medication to control HTN or cholesterol? 4) Have you smoked more than 100 cigarettes since becoming diabetic?

### HbA_1c_

Each individual was asked to provide a dried capillary blood spot which was collected using Roche Kit 14040. Blood glucose control was estimated by the analysis of the dried blood spot to determine each participant's current HbA_1c_ and was conducted by the Department of Clinical Chemistry, Skåne University Hospital SUS, Malmö, Sweden. There is excellent agreement (r = 0.99) between HbA_1c_ values from capillary blood on filter paper and HbA_1c_ values from venous blood [66].

### Retinal Photographs

A copy of the most recent fundus photographs were obtained from existing patient records. Records were collected from 79 clinics across Sweden. The photographs were graded by an experienced ophthalmologist [EA], blinded to baseline exposure status, using the International Clinical Diabetic Retinopathy Disease Severity Scale [67]. Retinopathy was defined as the presence of any of the following lesions: microaneurysms, retinal hemorrhages, hard or soft exudates or intra-retinal microvascular abnormalities. The primary outcome was the presence of any retinopathy based on fundus photos. If an individual had DR graded as questionable or photos were missing, DR classification from the NDR was used. In general, all the photos were 50° fields, taken through dilated pupils and stored as a digital image. Photos for 6 participants were slides, another 6 had either 45° or 60° fields and only 1 individual did not have their eyes dilated. In cases where multiple sets of photos were collected the most recent photos were graded and when both color and red-free photographs were available only the red-free photographs were used. Red-free photographs were available for 82% (193/235) of participants. Less than 7% (15/235) of individuals had photos with only 1 field centered on the fovea. When 2 fields were available, 1 was centered on the fovea and the other was either centered on the optic nerve or nasal to the optic nerve.

### National Diabetes Register

Supplemental information on retinopathy, HbA_1c_ and treatment for hypertension was provided by the National Diabetes Register (NDR). The NDR was implemented in 1996 by the Swedish Society for Diabetology as a response to the St. Vincent Declaration for Quality Assurance in Diabetes Care to survey the treatment and risk factor control in diabetic patients in everyday clinical practice [68]. Reporting to the NDR is based on information collected at least once a year during patient visits at hospital outpatient clinics and primary health centers all over Sweden. Data is supplied by trained nurses or physicians over the internet or by a preprinted form. Participation in this register is voluntary for the clinics and healthcare centers and all patients must be informed of the register and agree to participate. HbA_1c_ analyses are quality assured in Sweden. Both clinics and primary care centers use methods regularly calibrated with the HPLC Mono-S method.

The percent agreement and kappa of the measures of medication use for hypertension determined from the study questionnaire and the data supplied by the NDR was 86.5% and 0.65 respectively. The percent agreement and kappa for the outcome of the presence of DR in the study photos and the report supplied in the NDR was 78.0% and 0.55 for all the data and 86.5% and 0.72 for photos taken within 1 year of each other. The correlation between HbA_1c_ measures collected on filter paper and those reported in the NDR was 0.80.

### Statistical Analysis

Participants with type 2 diabetes were excluded from analyses. Baseline characteristics were reported for the individuals with type 1 diabetes and a reference group of non-diabetic controls including the percentage of males and participants initially treated with insulin; the mean and standard deviation of BMI and age at clinical diagnosis; and the median and inter-quartile range of C-peptide and diabetes autoantibody titers. Characteristics of individuals 15 years after the clinical onset of type 1 diabetes were also reported including the percentage of participants with dyslipidemia, kidney disease, treated HTN and who smoked more than 100 cigarettes since the clinical onset of diabetes. It also included the mean and standard deviation of glycosylated hemoglobin.

In the presence of a common outcome, such as DR in our study, point estimates for exposures associated with an increased risk of disease will be inflated using logistic regression. To eliminate this concern, we chose relative risk regression for our analyses. The Huber-White sandwich estimator of variance [69], [70] was specified.

Our primary analyses was restricted to four models fit to determine the independent associations of HLA, islet autoantibodies and C-peptide with DR. Model A was used to examine the crude association between HLA and DR unadjusted for other covariates except duration of diabetes. Any positive findings here would not indicate if the association between HLA and DR was independent or due to HLA's relationship with islet autoantibodies and C-peptide, Figure 1. Model B was used to examine the association between HLA and DR adjusting for precision variables and potential confounders [HbA_1c_ (%), age at diagnosis (years), sex and current use of hypertension medication (yes/no)]. In Model C, islet autoantibodies were added to Model B. This allowed the determination of the association between islet autoantibodies whether or not their effects were mediated through C-peptide. The fully adjusted Model D included HLA (DQ2, DQ8, DQ2/8 and DQ6), islet autoantibodies (GADA, ICA and IA-2A), C-peptide (nmol/l), age at diagnosis (years), sex, HbA_1c_ (%), treatment for hypertension (yes/no) and duration of diabetes (years). This model was used to determine the association of HLA with DR independent of islet autoantibodies and residual C-peptide. In addition it established the association of islet autoantibodies independent of residual C-peptide and adjusted for the potential confounding by HLA. Lastly this model showed the association of C-peptide with DR adjusted for confounding by HLA and islet autoantibodies.

Our secondary analyses examined the risk of retinopathy for subjects by the rate of change per year in GADA and C-peptide adjusting for baseline levels. By necessity these analysis were restricted to subjects with multiple measures. Since a number of subjects did not have multiple measures, this reduced the sample size and made comparisons of the risk of retinopathy between the primary analyses and the secondary analyses difficult. Therefore in the results from our secondary analysis, we included the findings from model D restricted to this smaller cohort. In Model E we looked at the risk of diabetic retinopathy using the last measure of GADA. In Model F we included the rate of change/year in GADA & C-peptide adjusting for initial levels. We have previously reported that GADA levels in GADA positive subjects remained unchanged after baseline [71] and very few participants with type 1 diabetes had measurable C-peptide by year 4 [28]. Due to the large confidence intervals for these estimates, particularly the change in C-peptide, we re-parameterized the model using tertiles of change in GADA/year and C-peptide/year. The reference group for the change in GADA/year was the group of subjects with the fastest decline in GADA/year and the reference group for C-peptide was the group with the slowest decline in C-peptide/year. Analyses were performed using Stata 8 (StataCorp. 2003. Stata Statistical Software: Release 8, StataCorp LP, College Station, TX).

## Results

We had complete data on 85% (246/289) of the participants with type 1 diabetes (Figure 2). Males comprised 55% (136/246) of the analytical group, compared to 67% males (219/329) in non-participants with type 1 diabetes (329/575) from the original cohort, p<0.01. GADA levels were 18 WHO units/ml higher in participants than non-participants, p = 0.05 but only 15 WHO units/ml higher, p = 0.10, after adjusting for sex differences. Participants and non-participants did not vary by C-peptide, IA-2A, ICA, BMI or age at diagnosis and had similar percentages of individuals who were HLA DQ2, 6 and 8; results not shown.

Most individuals had blood draws within the first month of diagnosis (89%). Median C-peptide levels were 0.27 nmol/l (Table 1) which was near the 2.5^th^ percentile of 829 age matched non-diabetic controls (0.24 nmol/l). Baseline characteristics of participants at the clinical onset of type 1 diabetes mellitus by HLA genotype are presented in Table 1 and by islet autoantibody status in Table 2. About 2/3^rd^ of participants with type 1 diabetes were DQ2, DQ8 or DQ2/8 while 4.9% (12/246) were DQ6. Mean HbA_1c_ was 7.0% 15 years later (Table 3). Characteristics of participants 15 years after the clinical onset of type 1 diabetes mellitus by HLA genotype are presented in Table 3 and by islet autoantibody status in Table 4. At that time, 15% reported they take medication for hypertension, 36% had dyslipidemia and 31% had been cigarette smokers at some time since developing type 1 diabetes. The median duration of diabetes was 15.2 years (Interquartile Range (IQR) 14.3–15.8).

**Table 1:** Characteristics of participants at the clinical onset of type 1 diabetes mellitus^a^ and reference panel of matched controls^b^ by HLA genotype.

		All Participants	DQ6	DQ8	DQ2	DQ2/8	Other HLA	Controls
Number	n (%)	246	12 (4.9)	62 (25.2)	39 (15.9)	59 (24.0)	74 (30.0)	837
Males	%	55.3	66.7	53.2	38.5	62.7	58.1	55.6
Age (years)	Mean (SD)	24.9 (5.4)	27.2 (5.4)	25.6 (5.1)	25.5 (5.4)	22.7 (5.2)	25.2 (5.3)	24.9 (5.9)
GADA (WHO units/ml)	Median (IQR)	99 (32–213)	57 (−2–262)	95 (28–194)	178 (58–252)	77 (27–170)	90 (44–239)	0 (0 - 0)
ICA (JDF-U)	Median (IQR)	54 (0–204)	108 (0–419)	84 (15–204)	54 (0–204)	54 (15–316)	54 (0–204)	0 (0 - 0)
IA-2A(WHO units/ml)	Median (IQR)	18 (0–262)	1 (−3–214)	200 (0–293)	0 (−3–9)	38 (0–235)	16 (0–266)	0 (0 - 0)
C-peptide (nmol/l)	Median (IQR)	0.27 (0.18–0.38)	0.29 (0.13–0.55)	0.27 (0.19–0.37)	0.26 (0.16–0.34)	0.29 (0.18–0.48)	0.28 (0.18–0.38)	0.59 (0.44–0.82)
BMI (kg/m^2^)	Mean (SD)	22.1 (3.7)	22.4 (3.7)	22.0 (3.4)	22.4 (4.4)	21.9 (3.2)	22.1 (3.9)	missing
Insulin Medication	%	88.9	75.0	87.1	89.7	94.7	87.8	0
Non-Diabetic reference	n (%)	837	234 (28.0)	141 (16.8)	141 (16.8)	24 (2.9)	297 (35.5)	

a Positive for GADA, ICA, IA-2A (first blood draw after diagnosis) or IAA (during the 1st month after diagnosis).

b These participants are a reference group of non-diabetics and were not used for analyses in the present study.

DQ6: DRB1*1501-DQA1*0102-B1*0602.

DQ8: DRB1*04-DQA1*0301-B1*0302.

DQ2: DRB1*0301-DQA1*0501-B1*0201.

HLA: human leukocyte antigen.

SD: standard deviation.

GADA: glutamic acid decarboxylase autoantibodies.

WHO: World Health Organization.

IQR: Inter-quartile range.

ICA: islet cell autoantibodies.

JDF-U: Juvenile Diabetes Foundation Units.

IA-2A: insulinoma antigen-2 autoantibodies.

BMI: body mass index.

**Table 2:** Characteristics of participants at the clinical onset of type 1 diabetes mellitus^a^ by islet autoantibody status.

		GADA+^b^	ICA+^c^	IA-2A+^d^
Number	n	203	177	141
Males	%	53.7	56.5	58.2
Age (years)	Mean (SD)	25.1 (5.3)	24.7 (5.5)	24.0 (5.3)
GADA (WHO units/ml)	Median (IQR)	130 (64–233)	122 (43–233)	98 (27–212)
ICA (JDF-U)	Median (IQR)	84 (0–204)	131 (54–316)	131 (35–316)
IA-2A(WHO units/ml)	Median (IQR)	12 (0–262)	144 (0–281)	247 (84–300)
C-peptide (nmol/l)	Median (IQR)	0.26 (0.18–0.36)	0.27 (0.18–0.37)	0.28 (0.17–0.42)
BMI (kg/m^2^)	Mean (SD)	21.9 (3.6)	22.2 (3.7)	21.9 (3.4)
Insulin Meds	%	91.0	89.7	92.1

a Positive for GADA, ICA, IA-2A (first blood draw after diagnosis) or IAA (during the 1^st^ month after diagnosis).

b glutamic acid decarboxylase autoantibody (GADA) positive >21.2 WHO units/ml.

c islet cell autoantibody (ICA) positive >6 Juvenile Diabetes Foundation Units.

d insulinoma antigen 2 autoantibody (IA-2A) positive >5.88 WHO units/ml.

SD: standard deviation.

WHO: World Health Organization.

IQR: Inter-quartile Range.

JDF-U: Juvenile Diabetes Foundation Units.

BMI: body mass index.

**Table 3:** Characteristics of participants 15 years after the clinical onset of type 1 diabetes mellitus^a^ by HLA genotype.

		All Participants	DQ6	DQ8	DQ2	DQ2/8	Other HLA
Number	n	246	12	62	39	59	74
HbA_1c_ (%)	Mean (SD)	7.0 (1.2)	6.6 (0.9)	7.0 (1.1)	7.2 (1.0)	6.9 (1.1)	7.1 (1.5)
HTN Meds^b^	%	15.0	8.3	21.0	18.0	10.2	13.5
Kidney Disease^b^	%	14.5	8.3	21.3	18.4	10.2	13.5
Dyslipidemia^b^	%	36.0	41.7	40.0	21.6	37.9	37.5
Smoker^c^	%	31.1	8.3	41.7	38.5	27.6	25.0

a Positive for GADA, ICA, IA-2A (first blood draw after diagnosis) or IAA (during the 1st month after diagnosis).

b Self-report, if missing NDR report.

c Smoked more than 100 cigarettes since the onset of diabetes.

HLA: human leukocyte antigen.

DQ6: DRB1*1501-DQA1*0102-B1*0602.

DQ8: DRB1*04-DQA1*0301-B1*0302.

DQ2: DRB1*0301-DQA1*0501-B1*0201.

SD: standard deviation.

HTN: hypertension.

**Table 4:** Characteristics of participants 15 years after the clinical onset of type 1 diabetes mellitus^a^ by islet autoantibody status.

		GADA+^b^	ICA+^c^	IA-2A+^d^
Number	n	203	177	141
HbA_1c_ (%)	Mean (SD)	7.0 (1.2)	7.0 (1.1)	6.9 (1.1)
HTN Meds^e^	%	16.3	17.5	13.5
Kidney Disease^e^	%	14.1	15.4	13.6
Dyslipidemia^e^	%	34.7	35.3	33.3
Smoker^f^	%	32.8	28.2	31.1

a Positive for GADA, ICA, IA-2A (first blood draw after diagnosis) or IAA (during the 1st month after diagnosis).

b glutamic acid decarboxylase autoantibody positive >21.2 WHO units/ml.

c islet cell autoantibody positive >6 Juvenile Diabetes Foundation Units.

d insulinoma antigen 2 autoantibody positive >5.88 WHO units/ml.

e Self-report, if missing NDR report.

f Smoked more than 100 cigarettes since the onset of diabetes.

The distribution of DR in graded photos was bimodal (Table 5). Based on the photos collected, 31.5% (74/235) of participants had no DR, however, 11 participants did not have photos and 24 were graded as questionable. After incorporating NDR data to classify these 35 individuals, the prevalence of any DR was 60.2% (148/246).

**Table 5:** Grade of retinopathy by eye from 235 participants with fundus photos.

	Right Eye		Left Eye		Both Eyes	
Grade	n	%	n	%	n	%
None	91	38.9	92	39.2	74	31.5
Mild	27	11.5	24	10.2	24	10.2
Moderate	92	39.3	87	37.0	107	45.5
Severe	3	1.3	2	0.9	4	1.7
PDR	2	0.9	2	0.9	2	0.9
Questionable	17	7.3	25	10.6	24	10.2
Unable to grade	2	0.9	3	1.3	0	0.0
Missing	11		11		11	

Relative risk regression models were used to determine the risk of retinopathy. The point estimate and 95% confidence intervals did not vary much between the four regression models (Table 6). In the fully adjusted Model D, increasing levels of GADA were associated with an increased risk of DR independent of HLA, C-peptide and known risk factors for DR including HbA_1c_ and hypertension, RR 1.12 per 100 WHO units/ml, [95% CI 1.02–1.23]. This yielded risk estimates of 1.27, [95% CI 1.04 to 1.62] and 1.43, [95% CI 1.06 to 1.94] for participants in the highest 25^th^ (GADA>233 WHO units/ml) and 5^th^ percentile (GADA>319 WHO units/ml) of GADA, respectively. In a similar model, classifying individuals as GADA positive (GADA>21.2 WHO units/ml) or negative, the risk for GADA positive participants at baseline was 1.37 [95% CI 0.98 to 1.92] compared to GADA negative individuals and using a slightly more strict definition for GADA positive (GADA>30.0 WHO unit/ml) the relative risk was 1.49 (1.10 to 2.01).

**Table 6:** Results of relative risk regression analyses for diabetic retinopathy, primary analysis.

	Model A		Model B		Model C		Model D	
n = 246	RR	95% CI	RR	95% CI	RR	95% CI	RR	95% CI
DQ2/8 (vs. other^a^)	0.88	0.67–1.15	0.91	0.70–1.20	0.91	0.70–1.19	0.92	0.70–1.19
DQ6 (vs. other^a^)	0.66	0.34–1.30	0.73	0.39–1.39	0.71	0.38–1.34	0.71	0.38–1.34
DQ8 (vs. other^a^)	1.03	0.81–1.31	1.03	0.81–1.30	1.07	0.85–1.34	1.06	0.84–1.34
DQ2 (vs. other^a^)	0.85	0.61–1.18	0.86	0.62–1.19	0.76	0.54–1.07	0.76	0.54–1.07
GADA (100 WHO units/ml)					1.12	1.02–1.23	1.12	1.02–1.23
log(ICA+0.1) (JDF-U)					1.01	0.98–1.04	1.01	0.98–1.04
IA-2A (100 WHO units/ml)					0.94	0.87–1.01	0.94	0.87–1.01
C-peptide (nmol/l)							0.95	0.67–1.33
HbA_1c_ (%)			1.16	1.07–1.25	1.16	1.07–1.26	1.16	1.07–1.26
HTN meds (yes vs. no)			1.38	1.12–1.70	1.36	1.11–1.67	1.37	1.11–1.68
Age at Diagnosis (years)			1.00	0.98–1.37	0.99	0.98–1.01	0.99	0.98–1.01
Males (vs. Females)			1.12	0.92–1.37	1.16	0.95–1.41	1.16	0.95–1.41
Duration (years)	1.13	1.02–1.26	1.11	1.01–1.22	1.11	1.02–1.22	1.11	1.02–1.22

a Other refers to anyone without DQ2, DQ8 or DQ6.

RR: relative risk.

DQ6: DRB1*1501-DQA1*0102-B1*0602.

DQ8: DRB1*04-DQA1*0301-B1*0302.

DQ2: DRB1*0301-DQA1*0501-B1*0201.

GADA: glutamic acid decarboxylase autoantibodies.

WHO: World Health Organization.

JDF-U: Juvenile Diabetes Foundation Units.

ICA: islet cell autoantibodies.

IA-2A: insulinoma antigen 2 autoantibodies.

The HLA haplotypes, DQ2, RR 0.76, [95% CI 0.54–1.07], DQ8, RR 1.06, [95% CI 0.84–1.34], DQ2/8, RR 0.92, [95% CI 0.70–1.19] and DQ6, RR 0.71, [95% CI 0.38–1.34], were not associated with the presence of any DR compared to participants who were not HLA DQ2, 8, 2/8 or DQ6. Likewise C-peptide, RR 0.95, [95% CI 0.67–1.33], ICA, RR 1.01, [95% CI 0.98–1.04] and IA-2A, RR 0.94, [95% CI 0.87–1.01] at the clinical onset of diabetes were not associated with DR.

In our secondary analyses, the risk of diabetic retinopathy was 1.12 (1.00–1.24) based on the last measure of GADA, slightly less than risk based on the first measure of GADA 1.15 (1.04–1.28) in the smaller sample size, Table 7. However, neither the rate of change of GADA/year (RR = 0.69, 0.16–3.04) or C-peptide/year (RR = 1.89, 0.20–17.6) was associated with the risk of diabetic retinopathy after adjusting for initial GADA and C-peptide level and the other covariates found in model D. Due to the large confidence intervals for the estimate of the risk of retinopathy for change in GADA/year and particularly the change in C-peptide, we re-parameterized the model using tertiles of change in GADA/year and C-peptide/year. The reference group for the change in GADA/year was the group of subjects with the fastest decline in GADA/year and the reference group for C-peptide was the group with the slowest decline in C-peptide/year. Rate of loss of GADA and C-peptide over the first six years after the clinical onset of diabetes was not associated with the risk of diabetic retinopathy 15 years later, Model F, Table 7.

**Table 7:** Results of relative risk regression analyses for diabetic retinopathy, secondary analyses.

	Model D		Model E		Model F	
n = 204	RR	95% CI	RR	95% CI	RR	95% CI
DQ2/8 (vs. other^a^)	0.84	0.61–1.15	0.80	0.59–1.11	0.84	0.62–1.15
DQ6 (vs. other^a^)	0.49	0.21–1.13	0.50	0.22–1.15	0.48	0.21–1.12
DQ8 (vs. other^a^)	1.01	0.77–1.32	1.00	0.77–1.31	1.01	0.78–1.32
DQ2 (vs. other^a^)	0.66	0.44–0.99	0.69	0.46–1.03	0.65	0.43–0.99
GADA (100 WHO units/ml)	1.15	1.04–1.28			1.16	1.04–1.30
Last GADA(100 WHO units/ml)			1.12	1.00–1.24		
Tertiles of loss of GADA/year						
Moderate loss of GADA vs. fastest					0.99	0.73–1.35
Slowest loss of GADA vs. fastest					0.90	0.69–1.17
log(ICA+0.1) (JDF-U)	1.01	0.98–1.05	1.02	0.98–1.05	1.01	0.98–1.05
IA-2A (100 WHO units/ml)	0.92	0.84–1.00	0.92	0.84–1.00	0.91	0.84–0.99
C-peptide (nmol/l)	0.88	0.62–1.27	0.89	0.60–1.32	0.88	0.62–1.26
Tertiles of loss of C-peptide/year						
Moderate loss of C-peptide vs. slowest					0.97	0.72–1.31
Fastest loss of C-peptide vs. slowest					0.99	0.75–1.30
HbA_1c_ (%)	1.20	1.10–1.32	1.20	1.09–1.32	1.21	1.09–1.32
HTN meds (yes vs. no)	1.37	1.08–1.74	1.38	1.08–1.76	1.36	1.06–1.75
Age at Diagnosis (years)	1.00	0.98–1.02	1.00	0.98–1.02	1.00	0.98–1.03
Males (vs. Females)	1.12	0.89–1.41	1.15	0.91–1.45	1.11	0.88–1.41
Duration (years)	1.10	0.99–1.23	1.11	0.99–1.24	1.09	0.98–1.22

a Other refers to anyone without DQ2, DQ8 or DQ6.

RR: relative risk.

DQ6: DRB1*1501-DQA1*0102-B1*0602.

DQ8: DRB1*04-DQA1*0301-B1*0302.

DQ2: DRB1*0301-DQA1*0501-B1*0201.

GADA: glutamic acid decarboxylase autoantibodies.

WHO: World Health Organization.

JDF-U: Juvenile Diabetes Foundation Units.

ICA: islet cell autoantibodies.

IA-2A: insulinoma antigen 2 autoantibodies.

## Discussion

This is the first study to report that increasing levels of GADA measured at the clinical onset of type 1 diabetes is associated with increased risk of DR after 15 years of follow-up. This association was independent of C-peptide, other islet autoantibodies, HLA DQ6, DQ2 and DQ8 as well as other major risk factors for DR including HbA_1c_ and hypertension. It is also of interest to note that even though GADA levels tend to be higher in subjects who are DQ2 (Table 1), the HLA haplotype DQ2 was not associated with diabetic retinopathy. This implies that the autoimmune response is more important in the risk of diabetic retinopathy than the immunogenetic association. In our secondary analyses, no associations were found between the change/year in GADA or C-peptide and the risk of diabetic retinopathy. The risk of retinopathy based on the last measure of GADA was 1.12 (1.00–1.24) was slightly attenuated compared to the risk based on the first measure of GADA 1.15 (1.04–1.28).

There are several strengths of this study. Our prospective study is composed of an incident inception population-based cohort and had a larger sample size than almost all other similar studies. We have much better measures of duration of diabetes than studies that rely on self-report. Our type 1 diabetes population is defined from laboratory measures of islet autoantibodies instead of physician classification. In addition, we have measures of islet autoantibodies and C-peptide at the clinical onset of diabetes which are not typically available in cross-sectional studies of DR.

One limitation of our study is that we only have a current HbA_1c_ which may not adequately reflect blood glucose control over the course of the study. Another possible limitation of our study is that the cohort consisted of participants who were between the ages of 15 and 35 at the time of clinical diagnosis of diabetes. Nevertheless, the cumulative prevalence of any DR in our cohort was about 60% after 15 years. This is similar to the prevalence of DR in other studies in Finland, Sweden and Wisconsin where the range of prevalence at 8 to 10 years duration of type 1 diabetes varied between 32 and 59% [72]. We had 2 participants (1%) with proliferative diabetic retinopathy; however, this estimate is likely too low. In a separate unpublished analysis of all patients receiving care at the Department of Ophthalmology in Malmö, Sweden, 8% (4/52) had PDR. This was among all patients with onset of diabetes <30 years of age and current duration of diabetes between 13 and 16 years. In addition, a previous study of a similar Swedish cohort [73] found that participants with worse DR were less likely to participate. This combined with our own unpublished analysis done in Malmö suggest participants with the worst retinopathy were less likely to participate in our study.

To date, there have been no studies linking islet autoantibodies with mechanisms leading to microvascular diseases. Of all the islet autoantibodies it seems more likely GADA may have some effect on the development of diabetic retinopathy since GAD65 is expressed in the neural retina as well as the pancreas and the central nervous system [14], [15], [16], [74]. GADA levels have been shown to remain elevated for many years after the clinical onset of diabetes [71], [75]. Among the islet autoantibodies only GADA have been linked to other clinical disease. For example, GADA has been implicated in differences in peripheral nerve function, independent of GADA related differences in glycemic control [76]. GADA is also a marker for Stiff-Person Syndrome [77] and bipolar disorder [78]. However, cause and effect relationships for these associations have not been demonstrated.

Two small cross-sectional studies have examined the relationship between GADA and DR. In one study (n = 80) [52], participants with less severe retinopathy were more likely to be GADA positive; 50%, 31% & 18% for non-DR, pre-PDR and PDR respectively. In another study (n = 55) GADA levels were lower in participants with severe disease compared to those without [51]. The observed inverse relationship between levels of GADA and the severity of DR in these two studies suggested that GADA may inhibit one or more mediators of DR. How GADA could inhibit DR is not clear. We hypothesize that if GADA is a factor, it would be in the progression of DR when the blood-retinal barrier is prominently compromised, allowing GADA access to antigen in the intra-retinal spaces, possibly modulating the inflammatory response. Our findings suggest an increased risk of DR for every 100 WHO units/ml increase in GADA (RR = 1.12) at the clinical onset of diabetes. However, our lack of participants with severe NPDR and PDR and different study designs make comparisons with these two previous studies difficult. One other study has reported no association between GADA and DR [79]. However the methodology of the study was flawed. Cases and controls were chosen by exposure status (24 GADA positive and 72 GADA negative subjects) instead of by the presence or absence of DR. This would severely limit the possibility of a positive finding.

There is a strong negative association between the DQ6 haplotype and type 1 diabetes among participants younger than 15 years of age [24]. However, the percentage of participants with the HLA DQ6 haplotype at clinical onset increases with increasing age and shows no association by 30–34 years of age [24]. In the present cohort, 4.9% (12/246) of participants were positive for HLA DQ6 which represents 14.0% (12/86) of participants not DQ2 or 8, and it is of considerable interest whether these participants might have a reduced risk for DR. Some researchers have discounted the HLA genes as a factor in the development and progression of DR due to the number of negative studies or the inconsistent results between studies. Previous studies were often hampered by the use of serologic or cellular typing of the HLA genes, had limited power, did not correct for multiple testing or did not adequately control for duration of diabetes, blood glucose levels and hypertension. However, at least one other study of individuals with younger-onset type 1 diabetes reported that the DQ6 haplotype was less common in participants with proliferative diabetic retinopathy (PDR) [45]. They found DQ6 in 6.7% (2/30) of participants with PDR compared to 14.0% (7/50) in participants with non-PDR and 12.0% (6/50) in healthy controls. In a comparison between the PDR group and the non-DR group they reported the odds of retinopathy was 0.4 for participants with DQ6 compared to those without. This is lower than our point estimate of 0.7, in a cohort consisting of much less severe DR.

We were unable to demonstrate an association between the amount of C-peptide at the onset of diabetes and the risk of DR 15 years later. We speculated that the patients with less C-peptide would be at greater risk for DR. In the much larger retrospective study from the DCCT, uniformly in the intensive and partially in the conventional treatment groups, any C-peptide secretion, but especially at higher and sustained levels of stimulated C-peptide, was associated with reduced incidences of DR, both a single three-step change and a repeated three-step change on the Early Treatment of Diabetic Retinopathy Study scale at the next 6-month visit [53].

It is not clear why our results differ from those seen in the DCCT. The DCCT did not consider islet autoantibody status. Another possibility is the difference in participants. To be eligible for the DCCT, patients were required to have had insulin dependent diabetes mellitus for one to five years and to have no retinopathy as detected by 7-field stereoscopic fundus photography. This is a more sensitive measure of retinopathy than we were able to attain in our sample. Their study period was also considerably shorter and measures of C-peptide more closely coincided with assessment of retinopathy. It may be possible that the absolute or nearly absolute loss of C-peptide increases the risk of diabetic microvascular complications and that the lower detection limit of our assay, 0.13 nmol/l, was not sensitive enough to distinguish these participants. The C-peptide binding curve to cell membranes of renal tubular cells, fibroblasts and endothelial cells indicate that saturation of binding occurs at very low concentrations [80], [81] possibly indicating very little C-peptide is needed to have the desired physiologic effect. It could also be that no C-peptide is a factor for patients with type 1 diabetes who develop retinopathy much earlier in the course of the disease. In our study, we do not know when DR first presented. If that information was available, Cox-regression could have been used to investigate whether C-peptide had a protective effect during the first years after the clinical onset of diabetes. Lastly, we cannot know from the DCCT or this study if C-peptide, endogenous insulin or both are potentially associated with DR.

Future studies will be needed to replicate these results and ideally these studies would include time to the onset of DR and a larger number of participants with more severe disease. Additional studies are needed to determine to what extent GADA may contribute to the immunofluorescence induced by serum samples tested on retinal cells [82], [83]. It is also of interest to determine if GADA interacts with other immunologic or metabolic factors to increase the risk of DR. Despite the number of previous studies of HLA and DR, there remains a need for a study with an adequate sample size to fully investigate these associations.

In conclusion, we have shown that increased levels of GADA at the time of onset were associated with an increased risk of DR 15 years later. These results, if confirmed, could provide additional insights into the pathogenesis of the most common microvascular complication of diabetes and lead to better risk stratification for both patient screenings and DR treatment trials.

## References

[1] (2008). Diabetes..

[2] (1993). The effect of intensive treatment of diabetes on the development
and progression of long-term complications in insulin-dependent diabetes
mellitus. The Diabetes Control and Complications Trial Research
Group.. N Engl J Med.

[3] (1998). Intensive blood-glucose control with sulphonylureas or insulin
compared with conventional treatment and risk of complications in patients
with type 2 diabetes (UKPDS 33). UK Prospective Diabetes Study (UKPDS)
Group.. Lancet.

[4] Keenan HA, Costacou T, Sun JK, Doria A, Cavellerano J (2007). Clinical factors associated with resistance to microvascular
complications in diabetic patients of extreme disease duration: the 50-year
medalist study.. Diabetes Care.

[5] Zhang L, Krzentowski G, Albert A, Lefebvre PJ (2001). Risk of developing retinopathy in Diabetes Control and
Complications Trial type 1 diabetic patients with good or poor metabolic
control.. Diabetes Care.

[6] Kupila A, Muona P, Simell T, Arvilommi P, Savolainen H (2001). Feasibility of genetic and immunological prediction of type I
diabetes in a population-based birth cohort.. Diabetologia.

[7] Rewers M, Bugawan TL, Norris JM, Blair A, Beaty B (1996). Newborn screening for HLA markers associated with IDDM: diabetes
autoimmunity study in the young (DAISY).. Diabetologia.

[8] Ziegler AG, Hillebrand B, Rabl W, Mayrhofer M, Hummel M (1993). On the appearance of islet associated autoimmunity in offspring
of diabetic mothers: a prospective study from birth.. Diabetologia.

[9] Bingley PJ, Christie MR, Bonifacio E, Bonfanti R, Shattock M (1994). Combined analysis of autoantibodies improves prediction of IDDM
in islet cell antibody-positive relatives.. Diabetes.

[10] Bonifacio E, Bingley PJ, Shattock M, Dean BM, Dunger D (1990). Quantification of islet-cell antibodies and prediction of
insulin-dependent diabetes.. Lancet.

[11] Bottazzo GF, Florin-Christensen A, Doniach D (1974). Islet-cell antibodies in diabetes mellitus with autoimmune
polyendocrine deficiencies.. Lancet.

[12] Lernmark A, Freedman ZR, Hofmann C, Rubenstein AH, Steiner DF (1978). Islet-cell-surface antibodies in juvenile diabetes
mellitus.. N Engl J Med.

[13] MacCuish AC, Irvine WJ, Barnes EW, Duncan LJ (1974). Antibodies to pancreatic islet cells in insulin-dependent
diabetics with coexistent autoimmune disease.. Lancet.

[14] Karlsen AE, Hagopian WA, Grubin CE, Dube S, Disteche CM (1991). Cloning and primary structure of a human islet isoform of
glutamic acid decarboxylase from chromosome 10.. Proc Natl Acad Sci U S A.

[15] Karlsen AE, Hagopian WA, Petersen JS, Boel E, Dyrberg T (1992). Recombinant glutamic acid decarboxylase (representing the single
isoform expressed in human islets) detects IDDM-associated 64,000-M(r)
autoantibodies.. Diabetes.

[16] Baekkeskov S, Aanstoot HJ, Christgau S, Reetz A, Solimena M (1990). Identification of the 64 K autoantigen in insulin-dependent
diabetes as the GABA-synthesizing enzyme glutamic acid
decarboxylase.. Nature.

[17] Lan MS, Lu J, Goto Y, Notkins AL (1994). Molecular cloning and identification of a receptor-type protein
tyrosine phosphatase, IA-2, from human insulinoma.. DNA Cell Biol.

[18] Payton MA, Hawkes CJ, Christie MR (1995). Relationship of the 37,000- and 40,000-M(r) tryptic fragments of
islet antigens in insulin-dependent diabetes to the protein tyrosine
phosphatase-like molecule IA-2 (ICA512).. J Clin Invest.

[19] Rabin DU, Pleasic SM, Shapiro JA, Yoo-Warren H, Oles J (1994). Islet cell antigen 512 is a diabetes-specific islet autoantigen
related to protein tyrosine phosphatases.. J Immunol.

[20] Palmer JP, Asplin CM, Clemons P, Lyen K, Tatpati O (1983). Insulin antibodies in insulin-dependent diabetics before insulin
treatment.. Science.

[21] Wenzlau JM, Juhl K, Yu L, Moua O, Sarkar SA (2007). The cation efflux transporter ZnT8 (Slc30A8) is a major
autoantigen in human type 1 diabetes.. Proc Natl Acad Sci U S A.

[22] Rolandsson O, Hagg E, Janer M, Rutledge E, Gaur LK (2003). High GAD65 autoantibody levels in nondiabetic adults are
associated with HLA but not with CTLA-4 or INS VNTR.. J Intern Med.

[23] Barker JM, Barriga KJ, Yu L, Miao D, Erlich HA (2004). Prediction of autoantibody positivity and progression to type 1
diabetes: Diabetes Autoimmunity Study in the Young (DAISY).. J Clin Endocrinol Metab.

[24] Graham J, Hagopian WA, Kockum I, Li LS, Sanjeevi CB (2002). Genetic effects on age-dependent onset and islet cell
autoantibody markers in type 1 diabetes.. Diabetes.

[25] Schenker M, Hummel M, Ferber K, Walter M, Keller E (1999). Early expression and high prevalence of islet autoantibodies for
DR3/4 heterozygous and DR4/4 homozygous offspring of parents with Type I
diabetes: the German BABYDIAB study.. Diabetologia.

[26] Pugliese A, Gianani R, Moromisato R, Awdeh ZL, Alper CA (1995). HLA-DQB1*0602 is associated with dominant protection from
diabetes even among islet cell antibody-positive first-degree relatives of
patients with IDDM.. Diabetes.

[27] Sherry NA, Tsai EB, Herold KC (2005). Natural history of beta-cell function in type 1
diabetes.. Diabetes.

[28] Jensen RA, Gilliam LK, Torn C, Landin-Olsson M, Karlsson FA (2007). Multiple factors affect the loss of measurable C-peptide over 6
years in newly diagnosed 15- to 35-year-old diabetic
subjects.. J Diabetes Complications.

[29] Agardh E, Gaur LK, Lernmark A, Agardh CD (2004). HLA-DRB1, -DQA1, and -DQB1 subtypes or ACE gene polymorphisms do
not seem to be risk markers for severe retinopathy in younger Type 1
diabetic patients.. J Diabetes Complications.

[30] Becker B, Shin DH, Burgess D, Kilo C, Miller WV (1977). Histocompatibility antigens and diabetic
retinopathy.. Diabetes.

[31] Groop LC, Teir H, Koskimies S, Groop PH, Matikainen E (1986). Risk factors and markers associated with proliferative
retinopathy in patients with insulin-dependent diabetes.. Diabetes.

[32] Hawrami K, Mohan R, Mohan V, Hitman GA (1991). A genetic study of retinopathy in south Indian type 2
(non-insulin-dependent) diabetic patients.. Diabetologia.

[33] Middleton D, Johnston PB, Gillespie EL (1985). HLA-DR antigen association with proliferative diabetic
retinopathy.. Int Ophthalmol.

[34] Mimura T, Amano S, Kato S, Araie M, Funatsu H (2004). HLA typing is not predictive of proliferative diabetic
retinopathy in patients with younger onset type 2 diabetes
mellitus.. Br J Ophthalmol.

[35] Weber B, Burger W, Hartmann R, Hovener G, Malchus R (1986). Risk factors for the development of retinopathy in children and
adolescents with type 1 (insulin-dependent) diabetes
mellitus.. Diabetologia.

[36] Wong TY, Cruickshank KJ, Klein R, Klein BE, Moss SE (2002). HLA-DR3 and DR4 and their relation to the incidence and
progression of diabetic retinopathy.. Ophthalmology.

[37] Agardh D, Gaur LK, Agardh E, Landin-Olsson M, Agardh CD (1996). HLA-DQB1*0201/0302 is associated with severe retinopathy in
patients with IDDM.. Diabetologia.

[38] Baker RS, Rand LI, Krolewski AS, Maki T, Warram JH (1986). Influence of HLA-DR phenotype and myopia on the risk of
nonproliferative and proliferative diabetic retinopathy.. Am J Ophthalmol.

[39] Birinci A, Birinci H, Abidinoglu R, Durupinar B, Oge I (2002). Diabetic retinopathy and HLA antigens in type 2 diabetes
mellitus.. Eur J Ophthalmol.

[40] Dornan TL, Ting A, McPherson CK, Peckar CO, Mann JI (1982). Genetic susceptibility to the development of retinopathy in
insulin-dependent diabetics.. Diabetes.

[41] Falck AA, Knip JM, Ilonen JS, Laatikainen LT (1997). Genetic markers in early diabetic retinopathy of adolescents with
type I diabetes.. J Diabetes Complications.

[42] Gray RS, Starkey IR, Rainbow S, Kurtz AB, Abdel-Khalik A (1982). HLA antigens and other risk factors in the development of
retinopathy in type 1 diabetes.. Br J Ophthalmol.

[43] Larkins RG, Martin FI, Tait BD (1978). HLA patterns and diabetic retinopathy.. Br Med J.

[44] Malone JI, Grizzard S, Espinoza LR, Achenbach KE, Van Cader TC (1984). Risk factors for diabetic retinopathy in youth.. Pediatrics.

[45] Mimura T, Funatsu H, Uchigata Y, Kitano S, Noma H (2003). Relationship between human leukocyte antigen status and
proliferative diabetic retinopathy in patients with younger-onset type 1
diabetes mellitus.. Am J Ophthalmol.

[46] Mimura T, Funatsu H, Uchigata Y, Kitano S, Shimizu E (2005). Gluatamic Acid Decarboxylase Autoantibody Prevalence and
Association with HLA Genotype in Patients with Younger-Onset Type 1 Diabetes
and Proliferative Diabetic Retinopathy.. Ophthalmology.

[47] Quiroz-Mercado H, Suarez-Licona A, Fromow-Guerra J, Lopez-Carasa G, Cardenas-Hernandez R (2002). Human lymphocyte antigen DR7 protects against proliferative
retinopathy with type II diabetes mellitus.. Arch Med Res.

[48] Rand LI, Krolewski AS, Aiello LM, Warram JH, Baker RS (1985). Multiple factors in the prediction of risk of proliferative
diabetic retinopathy.. N Engl J Med.

[49] Sterky G, Wall S (1986). Determinants of microangiopathy in growth-onset diabetes. With
special reference to retinopathy and glycaemic control.. Acta Paediatr Scand.

[50] Janeway CA, Travers P, Walport M, Shlomchik M (2005). Immunobiology the Immune System in Heatlh and Disease.

[51] Agardh D, Agardh E, Landin-Olsson M, Gaur LK, Agardh CD (1998). Inverse relationship between GAD65 antibody levels and severe
retinopathy in younger type 1 diabetic patients.. Diabetes Res Clin Pract.

[52] Mimura T, Funatsu H, Uchigata Y, Kitano S, Shimizu E (2004). Development and progression of diabetic retinopathy in patients
with Type 1 diabetes who are positive for GAD autoantibody.. Diabet Med.

[53] Steffes MW, Sibley S, Jackson M, Thomas W (2003). Beta-cell function and the development of diabetes-related
complications in the diabetes control and complications
trial.. Diabetes Care.

[54] Ostman J, Arnqvist H, Blohme G, Lithner F, Littorin B (1986). Epidemiology of diabetes mellitus in Sweden. Results of the first
year of a prospective study in the population age group 15–34
years.. Acta Med Scand.

[55] Littorin B, Sundkvist G, Schersten B, Nystrom L, Arnqvist HJ (1996). Patient administrative system as a tool to validate the
ascertainment in the diabetes incidence study in Sweden
(DISS).. Diabetes Res Clin Pract.

[56] Schranz DB, Bekris L, Landin-Olsson M, Torn C, Nilang A (1998). A simple and rapid microSepharose assay for GAD65 and ICA512
autoantibodies in diabetes. Diabetes Incidence Study in Sweden
(DISS).. J Immunol Methods.

[57] Olerup O, Zetterguist H (1993). DR “low-resolution” PCR-SSP typing–a correction
and an up-date.. Tissue Antigens.

[58] Olerup O, Zetterquist H (1992). HLA-DR typing by PCR amplification with sequence-specific primers
(PCR-SSP) in 2 hours: an alternative to serological DR typing in clinical
practice including donor-recipient matching in cadaveric
transplantation.. Tissue Antigens.

[59] Falorni A, Örtqvist E, Persson B, Lernmark Å (1995). Radioimmunoassays for glutamic acid decarboxylase (GAD65) and
GAD65 autoantibodies using ^35^S or ^3^H recombinant human
ligands.. J Immunol Methods.

[60] Verge CF, Gianani R, Kawasaki E, Yu L, Pietropaolo M (1996). Prediction of type I diabetes in first-degree relatives using a
combination of insulin, GAD, and ICA512bdc/IA-2
autoantibodies.. Diabetes.

[61] Torn C, Landin-Olsson M, Lernmark A, Palmer JP, Arnqvist HJ (2000). Prognostic factors for the course of beta cell function in
autoimmune diabetes.. J Clin Endocrinol Metab.

[62] Landin-Olsson M, Sundkvist G, Lernmark Å (1987). Prolonged incubation in the two-colour immunofluorescence test
increases the prevalence and titres of islet cell antibodies in type 1
(insulin-dependent) diabetes mellitus.. Diabetologia.

[63] Torn C, Landin-Olsson M, Lernmark Å, Palmer JP, Arnqvist HJ (2000). Prognostic Factors for the Course of {beta} Cell Function in
Autoimmune Diabetes.. J Clin Endocrinol Metab.

[64] Mueller PW, Bingley PJ, Bonifacio E, Steinberg KK, Sampson EJ (2002). Predicting type 1 diabetes using autoantibodies: the latest
results from the diabetes autoantibody standardization
program.. Diabetes Technol Ther.

[65] Verge CF, Stenger D, Bonifacio E, Colman PG, Pilcher C (1998). Combined use of autoantibodies (IA-2 autoantibody, GAD
autoantibody, insulin autoantibody, cytoplasmic islet cell antibodies) in
type 1 diabetes: Combinatorial Islet Autoantibody Workshop.. Diabetes.

[66] Jeppsson JO, Jerntorp P, Almer LO, Persson R, Ekberg G (1996). Capillary blood on filter paper for determination of HbA1c by ion
exchange chromatography.. Diabetes Care.

[67] Wilkinson CP, Ferris FL, Klein RE, Lee PP, Agardh CD (2003). Proposed international clinical diabetic retinopathy and diabetic
macular edema disease severity scales.. Ophthalmology.

[68] Gudbjornsdottir S, Cederholm J, Nilsson PM, Eliasson B (2003). The National Diabetes Register in Sweden: an implementation of
the St. Vincent Declaration for Quality Improvement in Diabetes
Care.. Diabetes Care.

[69] Huber PJ The behavior of maximum likelihood estimates under nonstandard
conditions; 1967.

[70] White H (1980). A heteroskedasticity-consistent covariance matrix estimator and a
direct test for heteroskedasticity.. Econometrica.

[71] Jensen R, Gilliam L, Torn C, Landin-Olsson M, Palmer J (2007). Islet cell autoantibody levels after the diagnosis of young adult
diabetic patients.. Diabet Med.

[72] McCulloch DK, Rose BD (2007). Pathogenesis and natural history of diabetic
retinopathy.. UpToDate.

[73] Henricsson M, Nystrom L, Blohme G, Ostman J, Kullberg C (2003). The incidence of retinopathy 10 years after diagnosis in young
adult people with diabetes: results from the nationwide population-based
Diabetes Incidence Study in Sweden (DISS).. Diabetes Care.

[74] Erdo SL, Wolff JR (1990). gamma-Aminobutyric acid outside the mammalian
brain.. J Neurochem.

[75] Borg H, Gottsater A, Fernlund P, Sundkvist G (2002). A 12-year prospective study of the relationship between islet
antibodies and beta-cell function at and after the diagnosis in patients
with adult-onset diabetes.. Diabetes.

[76] Hoeldtke RD, Bryner KD, Hobbs GR, Horvath GG, Riggs JE (2000). Antibodies to glutamic acid decarboxylase and peripheral nerve
function in type 1 diabetes.. J Clin Endocrinol Metab.

[77] Rakocevic G, Raju R, Dalakas MC (2004). Anti-glutamic acid decarboxylase antibodies in the serum and
cerebrospinal fluid of patients with stiff-person syndrome: correlation with
clinical severity.. Arch Neurol.

[78] Padmos RC, Bekris L, Knijff EM, Tiemeier H, Kupka RW (2004). A high prevalence of organ-specific autoimmunity in patients with
bipolar disorder.. Biol Psychiatry.

[79] Balme M, McAllister I, Davis WA, Davis TM (2002). Retinopathy in latent autoimmune diabetes of adults: the
Fremantle Diabetes Study.. Diabet Med.

[80] Rigler R, Pramanik A, Jonasson P, Kratz G, Jansson OT (1999). Specific binding of proinsulin C-peptide to human cell
membranes.. Proc Natl Acad Sci U S A.

[81] Wahren J, Ekberg K, Johansson J, Henriksson M, Pramanik A (2000). Role of C-peptide in human physiology.. Am J Physiol Endocrinol Metab.

[82] Agardh E, Bruun A, Ehinger B, Ekstrom P, van Veen T (1987). Gamma-aminobutyric acid- and glutamic acid
decarboxylase-immunoreactive neurons in the retina of different
vertebrates.. J Comp Neurol.

[83] Agardh E, Ehinger B, Wu JY (1987). GABA and GAD-like immunoreactivity in the primate
retina.. Histochemistry.

